# A neuroimaging point of view on the diversity of social cognition: evidence for extended influence of experience- and emotion-related factors on face processing

**DOI:** 10.1007/s40167-016-0043-6

**Published:** 2016-10-03

**Authors:** Nathalie George

**Affiliations:** 1 Social and Affective Neuroscience Laboratory and Centre MEG-EEG, Institut du Cerveau et de la Moelle épinière (ICM), CNRS / Inserm / UPMC, UMR7225 / U1127, GH Pitié-Salpêtrière, 47 bd de l’hôpital, 75013 Paris, France; 2 Sorbonne Universités, UPMC Univ Paris 06, UMR_S 1127, Paris, France; 3 CNRS, UMR 7225, Paris, France; 4 Inserm, U 1127, Paris, France; 5 ENS, Centre MEG-EEG, Paris, France

**Keywords:** Faces, Neuroimaging, MEG–EEG, Past experience, Emotion

## Abstract

Faces are key social stimuli that convey a wealth of information essential for person perception and adaptive interpersonal behaviour. Studies in the domain of cognitive, affective, and social neuroscience have put in light that the processing of faces recruits specific visual regions and activates a distributed set of brain regions related to attentional, emotional, social, and memory processes associated with the perception of faces and the extraction of the numerous information attached to them. Studies using neuroimaging techniques such as functional magnetic resonance imaging (fMRI) have allowed localizing these brain regions and characterizing their functional properties. Magnetoencephalography (MEG) and electroencephalography (EEG) techniques are complementary to fMRI in that they offer a unique insight into the temporal dynamics of mental processes. In this article, I review the contribution of neuroimaging techniques to the knowledge on face processing and person perception with the aim of putting in light the extended influence of experience-related factors, particularly in relation with emotions, on the face processing system. Although the face processing network has evolved under evolutionary selection pressure related to sociality-related needs and is therefore highly conserved throughout the human species, neuroimaging studies put in light both the extension and the flexibility of the brain network involved in face processing. MEG and EEG allow in particular to reveal that the human brain integrates emotion- and experience-related information from the earliest stage of face processing. Altogether, this emphasizes the diversity of social cognitive processes associated with face perception.

## Introduction: faces are rich social stimuli

The development of social processes is a central feature of human evolution, which may have had a foremost influence on human brain cortical expansion and associated cognitive development (Dunbar 1998; Barrett et al. 2003). The development of sociality may also have influenced the evolution of the human face. The human face has evolved to become not only the place where the organs of smell, taste, sound production, audition, and vision are gathered, but also an essential source of information for others. This took place in relation with the acquisition of bipedal locomotion, which has placed the face in a fully erected position, and in parallel with the development of articulated language.

The face primarily conveys persons’ identity: We are identified as individuals by our face (e.g., Bruyer 1987). Very importantly for social interactions, it also conveys some “generic identity” information, which is very important for social interactions. For example, information about the social categories to which we belong, such as our gender, age, ethnical categories, is conveyed by faces (Macrae and Quadflieg 2010). Social category learning is an important feature of human development, and most likely entails statistical learning about the links between some physical properties of the faces and some social categories (for example, wrinkled faces can be categorized as the faces of old persons). This is important because social categories gather information about the characteristics (that is, personality traits, taste, attitude, behavior, etc.) of other persons. For example, the “old persons” category is usually associated with the characteristics of being highly competent and experienced but also of being cognitively little flexible and potentially slow. Social categories form the backbone of stereotypes. Social categorical knowledge is acquired through experience or transmitted culturally. It plays a key role in impression formation on others. It contributes to our internal representations of others, shaping how we construe others and their behavior, our expectancies, and ultimately our interactions with others (Macrae and Bodenhausen 2000; Quinn et al. 2003).

In a cleverly designed study, Verosky and Todorov (2010) have recently shown evidence for the importance of past experience in impression formation on newly encountered persons. In this study, participants learned faces associated with positive, negative, and neutral behavior. Then, these faces were morphed with new faces, creating face blends containing 35 % of the learnt face. Importantly, these morphed faces were perceived by the participants as unfamiliar and were considered to resemble the learnt faces as little as totally new faces. In spite of this, when participants were asked to rate the morphed faces according to trustworthiness, their evaluation was biased as a function of the emotional valence of the behaviors associated with the original, learnt faces: the faces morphed with a face learnt in association with a positive behavior were judged as more trustworthy than the faces morphed with a face learnt in association with a negative behaviour. In others words, there was a learning generalization, with the evaluation of morphed faces being modulated by the valence of the behavior associated with the learnt face. Learning mechanisms based on similarity or regularity can therefore dynamically shape impression formation on faces and newly encountered others. This illustrates the plasticity of the face perception system at the behavioural level. In the next parts of this article, we will see how neuroimaging studies have allowed documenting the neural underpinnings of these processes and the wide extent to which experience-related (including emotional-experience-related) factors can modulate the brain responses to faces.

Faces also allow extracting information about the mental states of others—their objects of interest, how they feel,…—through the processing of emotional expression and gaze. This will not be much developed here because it is not at the core of this review. My aim will only be to illustrate the richness and the diversity of the processes triggered by face perception. The human face is (mostly) nude, it has a richly innervated musculature, which allows not only for speech production but also for the variety of facial expressions (for a review, see George 2013). While we share some prototypical patterns of facial expressions of emotions with other mammal species, particularly primates, the variety and the richness of emotional expressions seems unique to humans. Moreover, it seems too restrictive to limit the link between face and emotion to the production and perception of emotional expressions, because in fact emotions seems to be quite easily or automatically attached to even “neutral” individual faces (and they can potentially be generalized across individuals sharing some vague physical traits; see above). Accordingly, as we will see below, emotion-association and more generally emotional experience is an important factor of the flexibility of the brain network for face processing.

The eye region forms a key region of the face in relation with social cognitive processes and non verbal communication. The human eyes have evolved so that they have a specific elongated shape, with an extended white sclera, which make them not only the organ of vision but also an organ of communication with others (Kobayashi and Kohshima 1997, 2001). In brief, the direction of gaze gives invaluable indication about the direction of attention to others. When we see someone gazing at a surrounding location, we cannot help from shifting our spatial attention in the same direction (for a review of the experimental evidence for this “gaze cueing effect”, see Frischen et al. 2007). This interpersonal attention alignment phenomenon is at the basis of joint attention, which in turn is a cornerstone of our “mentalizing” capacity (Baron-Cohen 1995). Joint attention is the capacity to jointly attend with others to surrounding objects and it is believed to be a precondition to our capacity to mentalize, that is, to infer the state of mind (that is, the desires, preferences, beliefs, and thoughts) of others. Moreover, among all gaze directions, direct gaze, that is, gaze directed at oneself—which creates eye contact, holds a special status (for reviews, see Kleinke 1986; George and Conty 2008). It is the most basic and primary form of social contact. It is a preliminary to social interaction in adults, and it can have various meanings depending on context and culture: it can be evaluative, it can signal dominance or intimacy; it also plays a key role in the dynamic regulation of social exchanges (for example, during conversation) (Patterson 1982, 2011). Accordingly, as we will see below, the processing of gaze direction, including gaze contact, activates a wide set of brain regions related to attention, emotion, and mental state attribution.

## Neural underpinnings of face processing: on the extent and the flexibility of the face processing brain system

The development of neuroimaging studies has allowed identifying the brain regions involved in face processing. They form a distributed set of regions that encode the various information conveyed by faces (Haxby et al. 2000, 2002; Ishai 2008). Over the past 25 years, this network has been extensively studied and characterized. It comprises posterior brain regions that are considered to form the core face processing. These regions include the inferior occipital gyrus (IOG), the lateral fusiform gyrus (lFG), and the posterior superior temporal sulcus (pSTS). They are involved in the perceptual analysis of invariant—identity-related—(lFG) and variant—gaze, expression, and speech-related—face information (pSTS) (Hoffman and Haxby 2000). The role of the IOG is less clearly defined. It may be involved in the initial stages of face detection and visual encoding and it has recently been shown to be a region the lesion of which can be associated with prosopagnosia [a neuropsychological deficit characterized by a selective deficit in the recognition of faces that were previously known to the patient; (Bodamer 1947; Schiltz et al. 2006)]. Other regions form the extended face processing network (Haxby et al. 2000, 2002). They comprise posterior parietal regions involved in spatial attention, which are typically put in play during gaze processing, and regions of the emotional brain such as the amygdala, the insula, the ventral striatum, the orbitofrontal cortex, and other limbic or limbic-system-related regions, which are activated in particular for emotional expression processing. They also comprise a set of regions involved in person knowledge (Gobbini and Haxby 2007). These regions include the anterior paracingulate cortex and the dorsal medial prefrontal cortex (MPF), which are involved in the coding of personal traits, attitudes, and mental states. These regions act in concert with the temporo-parietal junction (TPJ) close to the pSTS region to decipher intentions and mental states in the course of social interaction (e.g., Pelphrey et al. 2003, 2004). Anterior temporal regions are involved in the memory for personal identity, name, and biographical information, and their superior lateral part would be more specifically involved in the neural representation of social concepts and social semantic knowledge (Zahn et al. 2007). More posterior regions of the precuneus and posterior cingulate cortex are involved in episodic aspects of the memories of persons, while inferior frontal gyrus regions are involved in semantic aspects of these memories (Gobbini and Haxby 2007; Ishai 2008). This widely distributed set of regions act in concert with other brain systems, for example those involved in memory formation (such as the hippocampus; Conty and Grezes 2012) and in the regulation of behavior (dorsal and ventral lateral prefrontal cortex, anterior cingulate cortex; Ito and Bartholow 2009) to form impression on persons and construe others.

The face is a key stimulus in social interaction and the extraction of the wealth of information that faces convey is essential for adaptive interpersonal behavior and navigating the social world (Bodenhausen and Macrae 2006). For this reason, one might think that the face processing brain network has developed in the course of human evolution and that it is rigidly engraved in the modern human brain. While the first part of this sentence is true, the second part obstructs the fact that face processing undergoes development to great extent throughout childhood and remains highly plastic all through the life span, thus adapting dynamically on the basis of past and ongoing experiences and interactions with others. Accordingly, the pattern of activation obtained during face and person processing tasks depends on context, subject’s goal, cognitive demands of the task, and idiosyncratic past experience (Ishai 2008). For example, passively viewing personally familiar faces as compared to famous faces (usually known more impersonally) or unknown faces activates the fusiform gyrus, the anterior paracingulate cortex, the precuneus, and the posterior superior temporal sulcus. On the contrary, it seems to elicit reduced amygdala activation (Gobbini et al. 2004; Gobbini and Haxby 2006). This indicates that there is an automatic retrieval of episodic information related to the personally familiar faces. In other terms, visual appearance seems to be just one component of familiar face recognition; familiar face recognition also involves the retrieval of person knowledge and associated episodic and emotional memories and familiarity induces changes in the neural representations of faces beyond mere visual memory (Gobbini and Haxby 2007).

It has been shown that the changes in the neural representations of faces can occur quite rapidly through only a few encounters with individuals. Todorov et al. (2007) presented to healthy adult participants a hundred and twenty unfamiliar faces associated with positive, negative, or neutral behavior. The faces were presented just twice in association with a sentence describing those behaviors. Then, these faces and some new faces were shown again in isolation and brain responses to those faces were recorded with fMRI. This allowed showing that there was a spontaneous reactivation of the memory trace of the faces seen previously, with activations in the paracingulate cortex and the posterior and anterior superior temporal sulcus. The activations were stronger when the behaviors associated with the faces were explicitly remembered. Yet, they were statistically significant even when the participant did not retrieve this previously acquired person knowledge. Moreover, a distinctive pattern of activation in emotional brain regions was observed as a function of the type of behavior associated with the faces. The faces associated with a disgusting behavior elicited stronger activation in the anterior insula than the faces associated with an aggressive behavior did and this activation was equally strong whether or not the behavior was recalled. The anterior insula has been specifically involved in the processing of disgust (e.g., Phillips et al. 1997; Wicker et al. 2003). Altogether, these results support the view that even minimal emotionally-laden past experience affects the memory trace for faces and that the acquired affective person knowledge is spontaneously retrieved when the faces are encountered again, engaging specific brain circuits involved in person memory and emotion processing.

Previous experience and knowledge on persons can be idiosyncratic and related to individual experience with the persons. It can also be related to social categories and stereotypes attached to social groups and acquired through cultural transmission. These social categories can concern race or gender for example. Lateral fusiform gyrus regions and posterior cingulate cortex were shown to be more activated in response to racial ingroup than racial outgroup faces (for a review, see Ito and Bartholow 2009). This could reflect greater familiarity with the ingroup than the outgroup faces. Yet it also suggests that outgroup faces may be processed less individually and less deeply, possibly reinforcing the application of stereotypic knowledge and eventually prejudice to outgroup individuals. Moreover, the activation of stereotypic thinking related to gender is associated with enhanced activation in an extended right frontal cortex region, suggesting that stereotype application during person perception draws upon brain regions involved in semantic memory about social categories (Mitchell et al. 2009). These brain responses were correlated with implicit attitude toward genders as revealed by the Implicit Association Test (IAT; Greenwald et al. 1998). In other words, the neural coding of faces and person is shaped by knowledge about social categories, which is acquired through social and cultural influences.

Interestingly, ingroup/outgroup processing biases can be dynamically modulated using for example a minimal group approach. Van Bavel et al. (2008) assigned participants randomly to mixed-race, arbitrarily formed teams and asked them to memorize the members of the teams. They found greater fusiform, amygdala and orbitofrontal responses to the “in-team” than the “out-team” member faces. Moreover, no difference in the brain responses to the faces of different races was found. Activation of the orbitofrontal cortex was correlated with greater liking of in-team than out-team faces. These results suggest that social motivation—and self-group or social category membership—may play a key role in the differentiated brain responses to ingroup versus outgroup faces. These brain responses underpin the mental representations of persons; they may subserve impression formation and influence how we interact with others. The social motivation process, as well as self-group membership, are quite flexible; they depend on culture, past experience (even when minimal), ongoing context, and subject’s goal.

In conclusion, neuroimaging studies emphasize the extent of the face processing system within the human brain, showing that it draws upon face specific visual analysis processes and upon general cognitive processes related to attention, emotion, motivation, and memory. They also demonstrate the flexibility of the face processing system and how it may be shaped by culture, socio-emotional processes, and personal past experience.

## Neural underpinnings of face processing: on the dynamics of face processing and how past experience and emotion affect the earliest stages of face processing

Another important question concerns the stages of face processing that may be permeable to the past experience of individuals, and therefore susceptible to be shaped by social and cultural influences. May the earliest stages of face processing be affected by past experience? Or is there only a late integration of past experience during face processing—after an initial stage of face visual analysis immune to ‘external’ influences—as postulated by classical models of face processing (Bruce and Young 1986; see also, Gobbini and Haxby 2007)? Functional brain imaging methods with a high temporal resolution, such as electroencephalography (EEG) and magnetoencephalography (MEG), which allow following the responses of neuronal assemblies at a millisecond timescale, are methods of choice to address this question.

We combined EEG and MEG techniques to investigate if emotional information may modulate subsequent traces for repeated stimuli from the earliest stages of face processing. In a first study, we examined the repetition effects for happy, fearful, and neutral faces (Morel et al. 2009). Repetition effects—that is, the differences in the responses to the first and subsequent presentations of a stimulus–are very useful to uncover the extent to which past experience modifies the current processing of a stimulus as function of different stimulus or context parameters. In this study, each face was repeated once, after 1–7 min and a minimum of 35 different faces between the first and the second presentation of a given face. This allowed us to reveal differentiated repetition effects for emotional and neutral faces from 40 to 50 ms after stimulus onset. These effects were also distributed over time, affecting not only the earliest visual response to the stimuli, but also the N170 and M170 components that peak between 150 and 200 ms and have been associated with the perceptual encoding of faces (e.g., Bentin et al. 1996; George et al. 1996; Rossion and Caharel 2011), and the M300 recorded in MEG around 300 ms. These results emphasized the great malleability of face processing by the human brain. They suggested that face processing can be modified from its earliest stage by experience-related and emotion-related factors.

Such very early emotion-related repetition effect may be associated with the processing of some low-level, coarse visual cues typical of the emotional expressions (e.g., local variations in the contrast around the mouth region produced by the smile for happy faces or wide sclera size of the fearful faces). However, in a follow-up MEG study, we used another paradigm, which allowed us to show that very early repetition effects can extend to associative emotional memory effects (Morel et al. 2012). In this paradigm, we used only neutral faces, which were associated with an emotional context on their first encounter. This context was an auditory verbal context; it described happy, anger-related, or neutral everyday life events that had happened to the person whose face was on display. Then the faces were seen again in isolation, between 1 and 7 min after their first occurrence and association with the emotional or neutral context. We found differentiated responses to the faces on the second encounter as early as between 30 and 60 ms after the face stimulus onset. In other words, the association of neutral faces with an emotional auditory-verbal context modulated the brain responses to these faces when they were encountered a second time, in isolation, and this modulation affected the earliest brain responses to the faces. The localization of the brain sources of this modulatory effect indicated the involvement of bilateral ventral occipito-temporal regions and right medial anterior temporal lobe regions. This finding reinforces the view of the brain as a highly malleable organ. It shows that the neural processing of faces can be shaped by experience from its earliest stage. It is also important to emphasize that, in this study, there was a single association of each face with either a happy, an anger-related, or a neutral context—with a total of 210 different faces and contexts shown. This suggests that the memory trace of faces integrates automatically contextual cues from even unique prior encounters.

One may wonder if the effects reported above pertain to the specific impact of emotions. Indeed, emotions relate to salient events or stimuli and they constitute adaptive responses of the organism to these events or stimuli, allowing the orientation of the priority of the individual toward the processing of the emotionally loaded information (see LeDoux 2012 for a recent review). They play a role in survival and it is therefore not surprising that they may have pervasive impact on stimulus processing. Yet, we have performed another study in which we examined the effect of social category associative learning on face processing (Gamond et al. 2011). In this study, we created a large set of different faces using a facial composite software available commercially. We manipulated systematically a physical feature of those faces, namely inter-eye distance, so that—while remaining in the normal range—the inter-eye distance was large for half the faces, and small for the other half. Then, the subjects were trained for about 20 min to categorize a subset of these faces as being those of either a determined or a flexible person. Of course, this task was totally artificial: There is no evidence that the flexible or determined nature of a person can be read solely from his/her physical facial features. However, humans are quite prone to infer personality traits from mere photographs of (even neutral) faces and the made-up association was here experimentally reinforced: Subjects received feedback on their response on each trial. Unbeknownst to them, we systematically associated the large (or small) inter-eye distance faces with either the ‘determined’ or the ‘flexible’ label. We performed recording of neuromagnetic responses to small and large inter-eye distance faces before and after this associative social category learning phase. This allowed us to show differentiated responses to large and small inter-eye distance faces as early as between 60 and 85 ms post-stimulus onset. This differentiated response was observed only *after* the experimentally induced association between inter-eye distance and social category labels; it was not observed before the reinforced associative learning phase. Hence, it was not due to the low level, physical difference between the large and small inter-eye distance faces. Source localization indicated the involvement of orbito-frontal and temporal lobe regions in parallel with more posterior inferior temporal regions of the ventral visual pathway. These findings supported the view that there is a very early interaction of prior experience with current sensory inputs, probably involving very early interaction between bottom-up and top-down feeds of information processing in anterior and posterior (sensory) regions of the brain. Notably, the potential affective connotations of the determined and flexible labels used here were carefully controlled and it is unlikely that the very early effect obtained in the study of Gamond et al. (2011) could be attributed to emotion-related effects. Altogether, this emphasizes the high degree of flexibility of the human brain, with a continuous adaptation of brain responses to incoming stimuli as a function of prior experience.

It is important to note a caveat to the studies mentioned above. The reported effects were not related to any behavioural outcome. That is, the studies used either incidental tasks unrelated to face emotion and repetition factors (Morel et al. 2009, 2012)—therefore not allowing to test for the potential behavioural influence of these factors—or an explicit task of social categorization that failed to demonstrate social category learning (Gamond et al. 2011). Therefore, it is important to emphasize that the functional role of the very early modulation of information processing in association with emotion, social category, and experience is unclear. It is likely that the earliest modulation observed are not directly related to behavioural outputs (that is, they may not influence directly how we evaluate faces and react to those faces). Yet, the results obtained suggest that the way we perceive others is fundamentally subjective, in the sense of being dependent on previous experience, which is moulded by emotional, social, and cultural factors.

Another important point to mention is that the processes of plasticity emphasized here may not be specific to face processing and person perception. First of all, a growing amount of studies have shown that emotions can have an impact on the earliest stages of stimulus processing, using non face stimuli (e.g., Stolarova et al. 2006). In the latter study, the authors showed that the C1 in response to simple gratings could be modulated when these gratings acquired an affective value through conditioning. Second, there is a vast literature on learning that shows that the human brain is exquisitely sensitive to statistical regularities and prone to learn categories (e.g., Sigala and Logothetis 2002), with the processes of learning impacting the earliest stages on stimulus processing (Chaumon et al. 2008, 2009). It is likely that social and cultural processes have evolved from existing characteristics of brain functioning, re-using fundamental properties of brain functioning (Dehaene and Cohen 2007), even if the development of social abilities may have been a driving force of cerebral expansion over the course of human evolution (Dunbar 1998).

## Conclusion

The human brain is highly flexible. This is a very general feature of brain function, which allows for extended learning and adaptation to variable environments. Yet, this malleability or flexibility may be particularly important when it comes to social processes and the perception of others. Human expertise in face perception is a universal feature of human cognition, which relies on highly specialized brain circuits that have developed through hominids evolution. Yet, it shows a high degree of variability across individuals as a function of their idiosyncratic experience, which is tightly related to the social group and cultural groups in which we grow up. This allows face processing and person perception to be shaped according to the diversity of social cognitive processes that may be brought into play depending on subject’s goal, on present contextual influence, and on past subject's experience.
